# Neospora caninum infection and repeated abortions in humans.

**DOI:** 10.3201/eid0502.990215

**Published:** 1999

**Authors:** E Petersen, M Lebech, L Jensen, P Lind, M Rask, P Bagger, C Björkman, A Uggla

**Affiliations:** Statens Serum Institut, Copenhagen, Denmark. ep@ssi.dk

## Abstract

To determine whether Neospora caninum, a parasite known to cause repeated abortions and stillbirths in cattle, also causes repeated abortions in humans, we retrospectively examined serum samples of 76 women with a history of abortions for evidence of N. caninum infection. No antibodies to the parasite were detected by enzyme-linked immunosorbent assay, immunofluorescence assay, or Western blot.


### *Neospora caninum* Infection and Repeated Abortions in Humans

**Eskild Petersen,* Morten Lebech,* Lene Jensen,† Peter Lind,† Martin Rask,† Peter Bagger,‡ Camilla Björkman,§ and Arvid Uggla§**
*Statens Serum Institut, Copenhagen, Denmark; †State Veterinary Laboratory, Copenhagen, Denmark; ‡Rigshospitalet, Copenhagen, Denmark; and §Swedish University of Agricultural Sciences, Uppsala, Sweden

To determine whether *Neospora caninum*, a parasite known to cause repeated abortions and stillbirths in cattle, also causes repeated abortions in humans, we retrospectively examined serum samples of 76 women with a history of abortions for evidence of *N. caninum* infection. No antibodies to the parasite were detected by enzyme-linked immunosorbent assay, immunofluorescence assay, or Western blot.

*Neospora caninum,* an intracellular protozoan parasite closely related to *Toxoplasma gondii* (1,2), was first described in dogs in Norway in 1984 and later in a wide range of other mammals including cattle, goats, horses, and sheep. The life cycle of *N. caninum* is only partially known, but the dog has recently been established as its definitive host (3). The pathogen's only known natural route of transmission (which can occur during sequential pregnancies in cattle) is transplacental (4).

*N. caninum* is now recognized as the most common cause of repeated abortions and stillbirths in cattle, and infected herds have been reported in most parts of the world, including Scandinavia (4-6). Infected, live-borne offspring may have neurologic symptoms including progressive paralysis. When experimentally transferred to pregnant nonhuman primates, *N. caninum* has caused fetal infection. The fetal lesions closely resembled those in congenital toxoplasmosis (7). *N. caninum* organisms are morphologically very similar to *T. gondii*, the pathogen responsible for toxoplasmosis; however, the two species have distinct antigenic characteristics and can be distinguished by serologic and immunohistochemical methods (4).

No case of *N. caninum* infection has been described in humans. However, because of the organism's close phylogenetic relationship to *T. gondii* and its wide range of potential hosts, the possibility of human *N. caninum* infection cannot be excluded. We investigated serologically the possible presence of *N. caninum* infection in Danish women who had repeated abortions of unknown cause.

Address for correspondence: Eskild Petersen, Laboratory of Parasitology, Statens Serum Institut, DK-2300 Copenhagen S. Denmark; fax:45-3268-3033; e-mail: ep@ssi.dk.

### The Study

The study included 76 women (mean age 30.8 years, range 19 to 41 years) who had had repeated abortions or intrauterine death of the fetus. Blood samples were obtained at the time of abortion or within 3 months of fetal death. The study participants had been referred to the Department of Gynecology and Obstetrics, Rigshospitalet, Copenhagen, Denmark, between 1 September 1991 and 31 October 1992 as part of a larger study of pregnant women with repeated primary or secondary abortions or repeated intrauterine fetal deaths. Serum specimens were tested for antibodies to *N. caninum* and *T. gondii* as described below.

### Findings

The absorbence values for the human serum samples were 0.10 to 1.24 absorbence units, whereas the mean value for the presumed *N. caninum*-negative human control serum was 0.26 (0.13 to 0.56). The mean absorbence values for the high-positive and low-positive control pig sera were 1.73 (1.54 to 1.93) and 0.87 (0.85 to 1.07), respectively. As no true *N. caninum*-negative or -positive human sera were available, serum specimens with absorbencies 0.50 (n = 12) were selected for further investigation (Table).

Table. Results of enzyme-linked immunosorbent assay (ELISA)[a] tests for *Neospora caninum* and of the Sabin-Feldman dye test[b] for *Toxoplasma gondii*

| Sample no. | ELISA mean OD | Dye test |
|---|---|---|
| 90 | 0.720 | 0 |
| 107 | 0.627 | 0 |
| 262 | 0.578 | 1:50 |
| 264 | 1.238 | 0 |
| 276 | 1.043 | 1:50 |
| 279 | 1.032 | 0 |
| 282 | 0.647 | 1:10 |
| 285 | 0.656 | 0 |
| 287 | 1.143 | 1:6250 |
| 289 | 0.818 | 0 |
| 295 | 0.583 | 0 |
| 297 | 0.541 | 0 |

[a]The ELISA was modified from Björkman et al. (8,9). A *T. gondii*-negative human serum specimen was used as a presumed *N. caninum*-negative control, and serum specimens from experimentally *N. caninum*-infected pigs were used as positive controls. A low-positive serum was collected from a pig infected 11 days before sampling and a high-positive control serum was pooled from pigs infected for at least 3 weeks (10). No reaction in human sera was definitely positive.

[b]The dye test was used to demonstrate antibodies to *T. gondii* as described (11) but using in vitro cultured *T. gondii*.

None of the 12 specimens showed specific fluorescence in the indirect fluorescence antibody test (IFAT) at dilution 1:640 with *N. caninum* tachyzoites that had been cultivated in vitro (6). (Sera had been diluted in twofold serial dilutions from 1:20 in phosphate-buffered saline.) Of the 12, only 3 had *T. gondii* antibodies. The reactivities in the *N. caninum* enzyme-linked immunosorbent assay (ELISA) were not associated with the presence of *T. gondii* antibodies (Table). Only 1 of the 12 human serum specimens tested showed reactivity against the *N. caninum* antigen by Western blot analysis. This specimen, number 279, recognized an antigen with apparent molecular weight of 60 kDa (Figure, lane 11 and 12). This antigen was not recognized by the *N. caninum*-positive pig sera, and serum 279 did not recognize any of the low-molecular weight antigens recognized by the *N. caninum*-positive pig sera. Three serum specimens reacted with the *T. gondii* antigen; all were *T. gondii*-positive in the dye test.

Because of the biologic similarities between *N. caninum* and the human pathogen *T. gondii*,

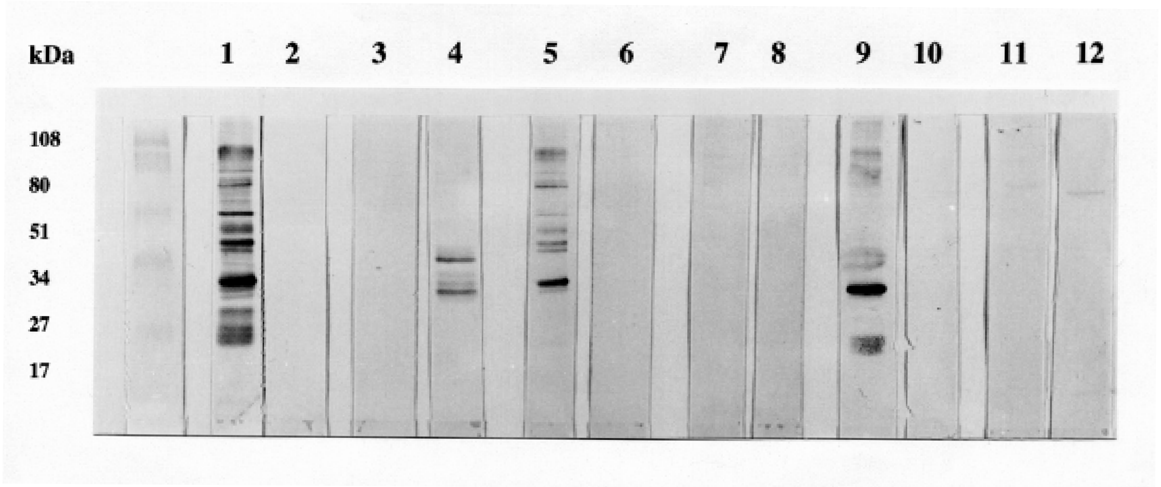

Figure. Western blot of *Toxoplasma gondii* or *Neospora caninum* antigen. Analysis was performed essentially as described by Sharma et al. (12) by using tachyzoites from in vitro culture of the *N. caninum* NC-1 isolate (13) and the *T. gondii* RH strain (10). Lanes 1-4 were probed with control sera and lanes 5-12 with human sera with high absorbencies in the *N. caninum* enzyme-linked immunosorbent assay.
Lane 1: *T. gondii*-positive human serum and *T. gondii* antigen; Lane 2: *N. caninum*-positive pig serum and *T. gondii* antigen; Lane 3: *T. gondii*-positive human serum and *N. caninum* antigen; Lane 4: *N. caninum*-positive pig serum and *N. caninum* antigen; Lane 5 and 6: Serum 262: *T. gondii* and *N. caninum* antigens, respectively; Lane 7 and 8: Serum 264: *T. gondii* and *N. caninum* antigens, respectively; Lane 9 and 10: Serum 276: *T. gondii* and *N. caninum* antigens, respectively. Lane 11 and 12: Serum 279: *T. gondii* and *N. caninum* antigens, respectively.

it has been speculated that *N. caninum* could be transmissible to humans. Since repeated abortions and stillbirths are common manifestations of neosporosis in cattle (4), women with a history of repeated abortions seemed an obvious category to investigate for human *N. caninum* infection. However, in this study of serum samples from women with repeated abortions, no evidence of *N. caninum* infection was detected.

The assays we used were based on methods used for *T. gondii* analyses; we used the same conjugates and serum dilutions found optimal in these analyses. The *N. caninum* immunostimulating complex antigen has a high specificity (14) and has been used for serologic investigations in different animal species (8,9,15). It was therefore anticipated that it would be applicable in a human system as well. However, because we could not define a proper cut-off for the assay, we further investigated the serum samples with the highest ELISA absorbence values by IFAT, regarded as the reference test for *N. caninum* antibodies in different species (4), and Western blot. None of the human sera investigated showed any reactivity in IFAT. Only one of the specimens reacted with the *N. caninum* antigen in the Western blot. However, because it only reacted with a band not recognized by sera from the infected pigs, the reaction was considered unspecific, and cross-reactivity between *T. gondii* and *N. caninum* was not found.

That we found no evidence of *N. caninum* infection in women who had repeated spontaneous abortions does not rule out the possibility that the infection might occur in humans. The predominant effects of neosporosis in dogs are primarily progressive neurologic signs including paralysis. It might, therefore, be worthwhile to examine human patients with clinical symptoms other than abortions, e.g., neurologic disorders of unknown etiology. Furthermore, the possible presence of *N. caninum* in patients with weakened immune systems should be considered. Researchers might continue the search for *N. caninum* by using serologic tests, as we did, or, alternatively, by using material collected at biopsy or autopsy for polymerase chain reaction or immunohistochemical analysis.

### Acknowledgments

We thank Lisbeth Petersen, Lis Wassmann, and Ann Lene Andresen for skillful technical assistance.

Dr. Petersen is a specialist in infectious diseases and tropical medicine at the Laboratory of Parasitology, Statens Serum Institut, Denmark's national reference center for diagnosis and research of human parasitic infections. His areas of expertise include immunology and epidemiology, primarily applied to malaria and congenital toxoplasmosis.

### References

1. Dubey JP, Carpenter JL, Speer CA, Topper MJ, Uggla A. Newly recognized fatal protozoan disease of dogs. J Am Vet Med Assoc 1988;192:1269-85.
2. Holmdahl OJM, Mattsson JG, Uggla A, Johansson K-E. The phylogeny of *Neospora caninum* and *Toxoplasma gondii* based on ribosomal RNA sequences. FEMS Microbiol Lett 1994;119:187-92.
3. McAllister MM, Dubey JP, Lindsay DS, Jolley WR, Wills RA, McGuire AM. Dogs are definitive hosts of *Neospora caninum*. Int J Parasitol 1998;28:1473-8.
4. Dubey JP, Lindsay DS. A review of *Neospora caninum* and neosporosis. Vet Parasitol 1996;67:1-59.
5. Holmdahl OJM, Björkman C, Uggla A. A case of *Neospora* associated bovine abortion in Sweden. Acta Vet Scand 1995;36:279-81.
6. Agerholm JS, Willadsen CM, Nielsen TK, Giese SB, Holm E, Jensen L, et al. Diagnostic studies of abortion in Danish dairy herds. J Vet Med 1997;A44:551-8.
7. Barr BC, Conrad PA, Sverlow KW, Tarantal AF, Hendrickx AG. Experimental fetal and transplacental *Neospora* infection in the nonhuman primate. Lab Invest 1994;71:236-42.
8. Björkman C, Lundén A, Holmdahl J, Barber J, Tress AJ, Uggla A. *Neospora caninum* in dogs: detection of antibodies by ELISA using an iscom antigen. Parasite Immunol 1994;16:643-8.
9. Björkman C, Holmdahl OJM, Uggla A. An indirect enzyme-linked immunosorbent assay (ELISA) for demonstration of antibodies to *Neospora caninum* in serum and milk of cattle. Vet Parasitol 1997;68:251-6.
10. Jensen L, Jensen TK, Lind P, Henriksen SA, Uggla A, Bille-Hansen V. Experimental porcine neosporosis. Acta Pathologica et Microbiologica Scandinavica 1998;106:475-82.
11. Sabin AB, Feldman HA. Dyes as microchemical indicators of a new immunity phenomenon affecting a protozoon parasite (*Toxoplasma*). Science 1948;108:660-3.
12. Sharma SD, Mullenax J, Araujo FG, Erlich HA, Remington JS. Western blot analysis of the antigens of *Toxoplasma gondii* recognized by human IgM and IgG antibodies. J Immunol 1983;131:977-83.
13. Dubey JP, Hattel AL, Lindsay DS, Topper MJ. Neonatal *Neospora caninum* infection in dogs: isolation of the causative agent and experimental transmission. J Am Vet Med Assoc 1988;193:1259-63.
14. Björkman C, Lundén A. Application of iscom antigen preparations in ELISAs for diagnosis of *Neospora* and *Toxoplasma* infections. Int J Parasitol 1998;28:187-93.
15. Huong LTT, Ljungström BL, Uggla A, Björkman C. Prevalence of antibodies to *Neospora caninum* and *Toxoplasma gondii* in cattle and water buffaloes in southern Vietnam. Vet Parasitol 1998;75:53-7.
